# Energetic cost of locomotion closely aligns with the preferred uphill walk–run transition at constant vertical speed in runners

**DOI:** 10.1007/s00421-026-06363-x

**Published:** 2026-07-30

**Authors:** Louis Finiel, Sophie Carron, Chloé Margot, Valentin Luc, Davide Malatesta, Fabio Borrani

**Affiliations:** Institute of Sport Sciences, Biomechanics & Integrative Physiology of Exercise Dynamics (BIPED) laboratory, University of Lausanne, Lausanne, 1015 Switzerland

**Keywords:** Mechanical work, Preferred transition speed, Locomotion, Uphill, Trail

## Abstract

**Purpose:**

The determinants of the preferred walk-to-run transition speed (PTS) during uphill locomotion remain unclear when the rate of vertical ascent is controlled rather than allowed to vary with treadmill speed. This study examined whether spatiotemporal, biomechanical, cardiorespiratory and energetic variables provide crossover-based optimal transition speed (OTS) that align with PTS during incline locomotion at constant vertical speed.

**Methods:**

17 Endurance-trained runners performed randomized six-minute walking and running bouts at speeds from 0.55 to 2.50 m·s⁻¹ for walking and from 0.55 to 3.88 m·s⁻¹ for running, all conducted at a constant vertical speed of 800 m·h⁻¹. The PTS assessment protocol was designed to maintain a constant rate of vertical ascent throughout the test.

**Results:**

The average PTS occurred at 1.76 m·s⁻¹ on a 7.2° slope. Energy and O_2_ cost of transport as well as metabolic power exhibited optimal transition speed that did not differ significantly from PTS, indicating a strong association between these energetic variables and the choice of gait. In contrast, heart rate, ventilation, and internal mechanical work displayed optimal transition speeds that were significantly different from PTS, while external and total mechanical work, step frequency, and step length showed no crossover between gaits.

**Conclusion:**

These findings indicate that, under conditions of constant vertical speed, uphill PTS aligns more closely with energetic crossover criteria than with cardiorespiratory or mechanical-work-based crossovers.

## Introduction

During steep uphill locomotion, the energetically less costly gait does not always correspond to the gait that individuals report they would spontaneously choose. For example, Giovanelli and coworkers showed, at a fixed vertical speed of 1260 m·h⁻¹, that metabolic power was lower during walking than running at inclines of 15.8° and above (Giovanelli et al. 2016). However, despite this energetic advantage of walking, participants reported that they would have chosen to run or alternate between walking and running if allowed to self-select their gait (Giovanelli et al. 2016). This discrepancy suggests that uphill gait selection cannot be fully understood from the energetic comparison of walking and running alone and highlights the need to investigate the mechanisms underlying the preferred transition speed (PTS), defined as the speed at which individuals spontaneously switch from walking to running.

Classical studies have established the energetic cost of walking and running across a wide range of gradients. Margaria first described the energetic behaviour of walking and running over different speeds and inclines, showing that walking can be energetically favoured over running at steep uphill gradients (Margaria 1938; Margaria et al. 1963). This framework was later extended by Minetti et al., who investigated extreme uphill and downhill slopes and confirmed that, at sufficiently high gradients, the energetic cost of locomotion is strongly related to the he vertical displacement of the body’s center of mass (Minetti et al. 2002). However, these studies primarily focused on the energetic cost of locomotion itself and were not designed to determine whether the spontaneous walk–run transition coincides with energetic or mechanical crossover criteria.

This issue was addressed more directly by Minetti et al., who investigated the transition between walking and running at different fixed gradients and found that PTS was lower than the oxygen-cost-of-transport optimal transition speed (O₂CoTOTS) up to 8.5° (Minetti et al. 1994a). More recently, Brill and Kram (2021) reported that, during uphill locomotion, PTS remained lower than the metabolic-power optimal transition speed (MPOTS) below 15°, before converging with it at this gradient. Together, these findings suggest that energetic criteria contribute to uphill gait transition, but that the preferred transition does not necessarily coincide with energetic optima under fixed-gradient conditions.

Other physiological variables may also contribute to the transition, although their role remains less clear. Heart rate (HR), despite being commonly monitored during uphill locomotion, has shown inconsistent associations with PTS and HR-based optimal transition speed (HROTS) (Brill and Kram 2021). Ventilation (V̇E) has received even less attention in this context, although uphill running modifies breathing patterns and may affect the synchronization between locomotor and respiratory rhythms, known as locomotor–respiratory coupling (Bramble and Carrier 1983; Takano 1995). Thus, whether a ventilatory crossover could align with PTS during steep uphill locomotion remains unknown. Overall, despite the contribution of previous energetic studies, there is still no clear consensus on which physiological variable best aligns with the preferred walk–run transition during uphill locomotion.

In uphill locomotion, external mechanical work (Wext) increases with steeper inclines, as greater positive work is required to elevate the body’s center of mass (CoM). This increased demand is primarily accommodated by greater force production from the rear lower limb during push-off, resulting in more positive mechanical work as the gradient rises, while negative work from the front lower limb progressively decreases (Franz et al. 2012; Dewolf et al. 2016, 2017). Previous studies measuring mechanical work during gradient locomotion have consistently shown that Wext represents a major component of the mechanical demand in steep uphill walking and running, thereby contributing substantially to the increase in total mechanical work (Wtot) with incline (Minetti and Ardigò 2001; Rasica et al. 2020; Luciano et al. 2024). In parallel, internal mechanical work (Wint), i.e., the work required to move the limbs relative to the CoM, also increases with changes in locomotor mechanics, particularly through modifications in stride frequency and limb segment motion (Minetti et al. 1994b; Minetti 1998; Nardello et al. 2011). Importantly, Wint is also strongly influenced by speed, and its increase with speed is steeper during walking than during running, which may amplify gait-specific differences in internal work near the walk–run transition (Minetti et al. 1994b). Thus, the concomitant increase in Wext and Wint during uphill locomotion may contribute to the rise in Wtot and metabolic cost as well as to the biomechanical constraints associated with the walk–run transition at steep gradients (Minetti et al. 1994a; Roberts and Belliveau 2005; Snyder and Farley 2011; Vernillo et al. 2017).

Most previous studies have used fixed-incline protocols in which treadmill speed is increased in steps to determine PTS (Hreljac 1993; Minetti et al. 1994a; Brill and Kram 2021). Although this approach provides a straightforward manipulation of belt speed, increasing speed at a given incline necessarily increases vertical ascent rate and therefore the mean net potential power (P_pot, net_) i.e., the average rate of net gravitational potential energy gain required to raise the body’s center of mass. In parallel, metabolic power typically increases with speed at a fixed grade. As a result, physiological responses and several kinematic descriptors tend to co-vary with the imposed speed increments, complicating the interpretation of associations with PTS. An iso-vertical-speed (iso-V_vert_) protocol, in which vertical speed is held constant while treadmill speed and incline are adjusted, constrains Ppot, net across conditions while still allowing gait-dependent mechanical variables, such as Wint, Wext, Wtot, and the distribution of positive and negative increments of mechanical energy, as well as physiological responses, to vary along the slope–speed contour. Because speed and grade are coupled by design, the aim is not to isolate their independent effects, but to evaluate which candidate determinants are most closely aligned with PTS under a controlled ascent-rate condition.

The aim of this study was to quantify PTS during uphill locomotion performed at a constant vertical speed and to compare PTS with variable-specific OTS values computed as the speed at which walking and running curves intersect. Based on prior fixed-slope work showing that PTS remains slower than energetic optima at inclines below ~ 15° and converges only at steeper inclines (Brill and Kram 2021), OTS derived from energetic criteria were expected to occur at faster speeds than PTS under the present ascent-rate condition. HROTS was expected to differ from PTS, whereas V̇EOTS was expected to coincide with PTS (i.e., not differ from PTS). Similarly, OTS derived from Wint was expected to not differ from PTS (i.e., WintOTS ≈ PTS), consistent with the idea that preferred gait selection may align with an internal-work-related criterion under uphill conditions. Step-frequency OTS was also hypothesized to coincide with PTS due to the simultaneous change in speed and slope. Finally, no OTS was expected for variables unlikely to exhibit a walk–run crossover within the tested range (Wext, Wtot, step length).

## Materials and methods

### Participants

17 male long-distance runners consented to participate in this investigation. The mean ± standard deviation (SD) values for age, body mass, and height were 25 ± 4 years, 68.8 ± 7.2 kg, and 1.80 ± 0.07 m, respectively. Inclusion criteria stipulated that participants must be able to complete a ten-kilometer run within thirty-eight minutes and have no medical conditions that could impact study outcomes or athlete health as confirmed by the Physical Activity Readiness Questionnaire (PAR-Q). Ethical approval for the study was obtained from the local ethics committee (VD No 2022–02068), and written informed consent was received from all participants. This research adheres to the principles outlined in the latest Helsinki Declaration on Human Experimentation (2013). Throughout the study period, participants were instructed to refrain from engaging in unusual training activities, and a minimum of 48 h was mandated between study sessions.

### Study design

The participants visited the laboratory on six occasions over a span of three to four weeks. The first visit involved familiarization with the laboratory setting and determination of preferred transition speed (PTS) on an incline treadmill prototype (Le mur, Issul Lab, Lausanne, Switzerland) specially designed to be able to change speed and automatically modify gradient to maintain a constant ascent rate. PTS was determined as the average of the walk-to-run and run-to-walk transition speeds at 800 m·h⁻¹ (0.22 m·s⁻¹) vertical speed (Hreljac et al. 2007; Brill and Kram 2021). The target vertical speed of 800 m·h⁻¹ was selected to represent a field-relevant uphill intensity for trained mountain runners. Publicly available performance data from verified UTMB uphill segments show that elite female records average approximately 788 m·h⁻¹ and elite male records approximately 975 m·h⁻¹ across major climbs, with several sustained ascents displaying record vertical speeds close to or above 800 m·h⁻¹. Therefore, 800 m·h⁻¹ can be considered a demanding but realistic ascent rate, representative of competitive uphill locomotion rather than of average race participation.

At this fixed vertical speed during the PTS determination test, each participant began at a treadmill speed of 1.11 m·s⁻¹. During walk-to-run transition speeds, speed increased by 0.2 km·h⁻¹ (0.056 m·s⁻¹) every 30 s. When the participant spontaneously switched gaits, the treadmill was stopped, and then they performed 30 s of walking followed by 30 s of running at that speed to confirm their final decision. If the participant was uncertain, the incremental test continued until a definitive transition speed was identified (Brill and Kram 2021). During the run-to-walk transition speed, the protocol was identical, but in reverse order, starting at a treadmill speed of 2.5 m·s⁻¹. The transition between the two types of locomotion was determined based on the participants’ sensations. Although the gaits are quite similar on uphill inclines, previous research suggests that participants can intuitively and easily distinguish between walking and running (Ortiz et al. 2017). Visit two to five each consisted of a subset of the 17 modalities, which included both walking and running trials performed on an inclined treadmill at a constant ascent rate of 800 m·h⁻¹. Eight travel speeds were tested for walking (0.56, 0.83, 1.11, 1.39, 1.67, 1.94, 2.22 and 2.50 m·s⁻¹) and nine for running (0.56, 0.97, 1.39, 1.81, 2.22, 2.65, 3.06, 3.47, 3.89 m·s⁻¹). See Table 1 for corresponding angles. Modality is defined as a 6-minute effort at a precise speed in one of the two locomotion (i.e., walking or running). Due to the uneven number of total modalities, one visit included five modalities and three included four. The assignment of modalities to each visit, as well as their order of presentation within a visit, was fully randomized before testing for each participant using a random sequence generator. No testing was conducted on Mondays, in consideration of common training habits among runners and trail runners, who often perform long or intense sessions during the weekend. A typical laboratory visit started with a 3-minute rest period, followed by a 6-minute warm-up on the treadmill at 2.78 m·s⁻¹ and a 2º slope. Subsequently, participants executed 5 or 4 modalities of 6-minute duration. A 6-minute passive rest was observed between each modality. During all tests, gas exchange was measured breath-by-breath using a portable metabolic system (Cosmed K5, Cosmed, Italy) and heart rate (HR) using a heart rate belt (Garmin HRM, U.S.A). The gas analyzers were calibrated before each test using the two known gas concentrations (ambient air and a mixture of 16% O_2_ and 5% CO_2_), to correct both slopes and offset errors. The volume sensor was calibrated using a 3-liter syringe (Hans Rudolph, Kansas City, MO). Finally, during the last visit, participants executed each of the 17 modalities for a duration of 30 s separated by breaks in order to avoid any fatigue to quantify kinematic parameters.

**Table 1:** Treadmill belt speed (m·s⁻¹), corresponding incline angle (°), vertical speed and locomotion mode tested at a constant vertical speed of 800 m·h⁻¹ (0.22 m·s⁻¹)

Speed (m·s⁻¹)	Angle (°)	Slope (%)	Vertical speed (m·h⁻¹)	Locomotion
0.56	23.58	43.65	800	W - R
0.83	15.45	27.64	800	W
0.97	13.21	23.47	800	R
1.11	11.53	20.40	800	W
1.39	9.20	16.20	800	W - R
1.67	7.66	13.45	800	W
1.81	7.07	12.40	800	R
1.94	6.56	11.50	800	W
2.22	5.74	10.05	800	W - R
2.50	5.10	8.92	800	W
2.64	4.83	8.45	800	R
3.06	4.17	7.29	800	R
3.47	3.66	6.40	800	R
3.89	3.27	5.71	800	R

Locomotion type is coded as “W” (walking) and “R” (running). “W–R” denotes speeds at which both gaits were tested/observed because walking and running were sampled using different speed increments (0.27 m·s⁻¹ for walking; 0.41 m·s⁻¹ for running) starting from 0.56 m·s⁻¹, resulting in overlapping speed levels.

### Data analysis

Variables from the gas analyzer, i.e. V̇O_2_, V̇E, $\:\dot{E}$ and HR, were first examined and erroneous respirations due to swallowing, sighing or coughing were excluded when the values deviated by more than three standard deviations from the local mean. The net energy rate (Ė, kcal·min⁻¹·kg⁻¹) was obtained from the COSMED K5 indirect calorimetry output, which derives energy expenditure from measured V̇O₂ and V̇CO₂ using the abbreviated Weir equation (implemented in the K5 software), and was then expressed as a mass-specific rate (Weir 1949).

Net oxygen cost of transport for walking in ml/kg/km was obtained using this equation:

$$ \:O_{2} CoT = \dot{V}O_{{2net}} /V\: $$

Where V̇O_2net_ is the net oxygen consumption in mlO_2_/min/kg and V is the speed associated expressed in km/min. For each physiological variable at each modality, the final minute of the 6-minute exercise period was averaged. Net Metabolic Power (MP) for walking and running are expressed in W·kg⁻¹ following the equation below:

$$ \:MP = \dot{E}net \cdot \:\:1000\: \cdot \:4.18678/60 \cdot \: $$

Where $\:\dot{E}$net is the net energy rate in kcal/min/kg multiplied by 1000 to have cal/min/kg. 4.18678 is the equivalent of cal in Joules and divided by 60 to transform into watts (J/s).

Additionally, the net energy cost of transport for walking and running is expressed in J·kg⁻¹·m⁻¹ and was obtained by the following equation:

$$ \:ECoT = \dot{E}net\:1000 \cdot \:4.18678/V \cdot $$

Where $\:\dot{E}$net is the net energy rate in kcal/min/kg multiplied by 1000 to have cal/min/kg, 4.18678 is the equivalent of cal in Joules and V is the treadmill speed in m/min.

Three-dimensional full-body kinematic data were recorded at a sampling rate of 200 Hz using an 11 cameras motion capture system (Vicon, Nexus software v2.9.3, Oxford, UK). A total of 43 reflective markers (diameter: 12.5 mm) were used for the static calibration trials, and 39 were used during dynamic locomotion trials. These markers were attached directly to the skin and shoes using adhesive tape, following established anatomical landmark placement protocols (Patoz et al. 2022). Kinematic data was saved in.c3d format and imported into Visual3D Professional (v2024.09.1, C-Motion Inc., Germantown, MD, USA) for processing. The data were then low-pass filtered at 20 Hz using a fourth-order Butterworth filter. A biomechanical model consisting of 15 rigid body segments and six degrees of freedom was built from the marker data. This model included the head, trunk, pelvis, upper (arms, forearms and hands) and lower (thighs, shanks, and feet) limbs. Segmental inertial properties and center of mass positions were assigned based on geometric approximations (Hanavan 1964) and standard anthropometric regression equations (Dempster 1955). The whole-body COM was derived from the combined segmental data and calculated directly within Visual3D. For analysis, biomechanical variables were extracted from a 30 s period of data collection for each participant. Both left and right step data were included and averaged to produce representative values used in the final statistical evaluations. From the CoM trajectory, instantaneous gravitational potential energy was computed as $\:{E}_{p}\left(t\right)=m{\hspace{0.17em}}g{\hspace{0.17em}} z(t)$, where $\:z\left(t\right)$is CoM vertical position, and instantaneous kinetic energy as $\:{E}_{k}\left(t\right)=\frac{1}{2}m\left({v}_{x}(t{)}^{2}+{v}_{y}(t{)}^{2}+{v}_{z}(t{)}^{2}\right)$, where $\:{v}_{x},{v}_{y},{v}_{z}$are the CoM velocity components derived by 3-axes CoM displacements. External mechanical work ($\:{W}_{ext}$) was then quantified using the “positive increments” method, as the sum of the positive changes in total CoM mechanical energy $\:{E}_{tot}\left(t\right)={E}_{p}\left(t\right)+{E}_{k}\left(t\right)$ over each step (Minetti et al. 1993). Internal mechanical work ($\:{W}_{int}$) was computed as the sum of positive changes in segmental kinetic energies relative to the body CoM (linked-segment approach), following established procedures (Willems et al. 1995). Total work was calculated as $\:{W}_{tot}={W}_{ext}+{W}_{int}$. Work values were subsequently normalized to body mass (and, when reported as work per distance covered during the analyzed window) to allow comparisons across participants and conditions.

Step frequency was calculated as the inverse of step time, which was defined as the duration between two successive peaks in the vertical displacement of the CoM along the z-axis. All values were averaged across steps recorded during the 30-second data collection period for each participant. Step length was computed by accounting for both the treadmill belt displacement and the participant’s anteroposterior position relative to a fixed reference point at each step cycle.

### Statistical analysis

For each participant and outcome variable, the relationship with speed was modelled using polynomial regression. Given the limited number of data points per condition (walking: *N* = 8; running: *N* = 9), polynomial order was capped at three to maintain parsimony and reduce the risk of overfitting. Within this constraint, models of increasing order (degree 1–3) were compared using the small-sample corrected Akaike Information Criterion (AICc) (Babyak 2004; Symonds and Moussalli 2011). A visual inspection of the residuals was performed in place of a homoscedasticity test (Knief and Forstmeier 2021; Katsileros et al. 2024). The OTS for each variable for each individual was defined as the point of intersection between the walking and running curves (if it existed).

A bootstrap resampling approach was employed to provide robust estimation of confidence intervals and statistical significance. Unlike traditional parametric methods such as ANOVA, linear mixed models, or t-tests, which rely heavily on assumptions of normality and homoscedasticity, bootstrap methods make no such distributional assumptions and are therefore particularly well suited to physiological and biomechanical data (MacDonald et al. 2024). This flexibility is especially valuable when sample sizes are modest, as is common in experimental research, where verifying distributional assumptions can be challenging (Efron and Tibshirani 1994; Chernick 2007). Bootstrap methods are well-regarded for their reliability under such conditions (Davison and Hinkley 1997; Bland and Altman 2015). The procedure involves resampling the original dataset with replacement to create multiple “bootstrap replicate” datasets, each of the same size as the original dataset. A random number generator is used to determine which data points from the original dataset will be included in each replicate. A given data point may appear multiple times or not at all in a replicate dataset. For each replicate, the PTS and OTS parameters of each variable are estimated using the same procedures applied to the original dataset. This process is repeated 1,000 times, and the parameter estimates are recorded. For each parameter of each variable, the difference between PTS and OTS was calculated across all resamples to derive the mean difference and the 2.5th and 97.5th percentiles, which defined the bounds of the 95% CI. A difference was considered statistically significant if the CI did not include zero. Data are reported as mean ± 95% CI.

## Results

**Fig. 1:**
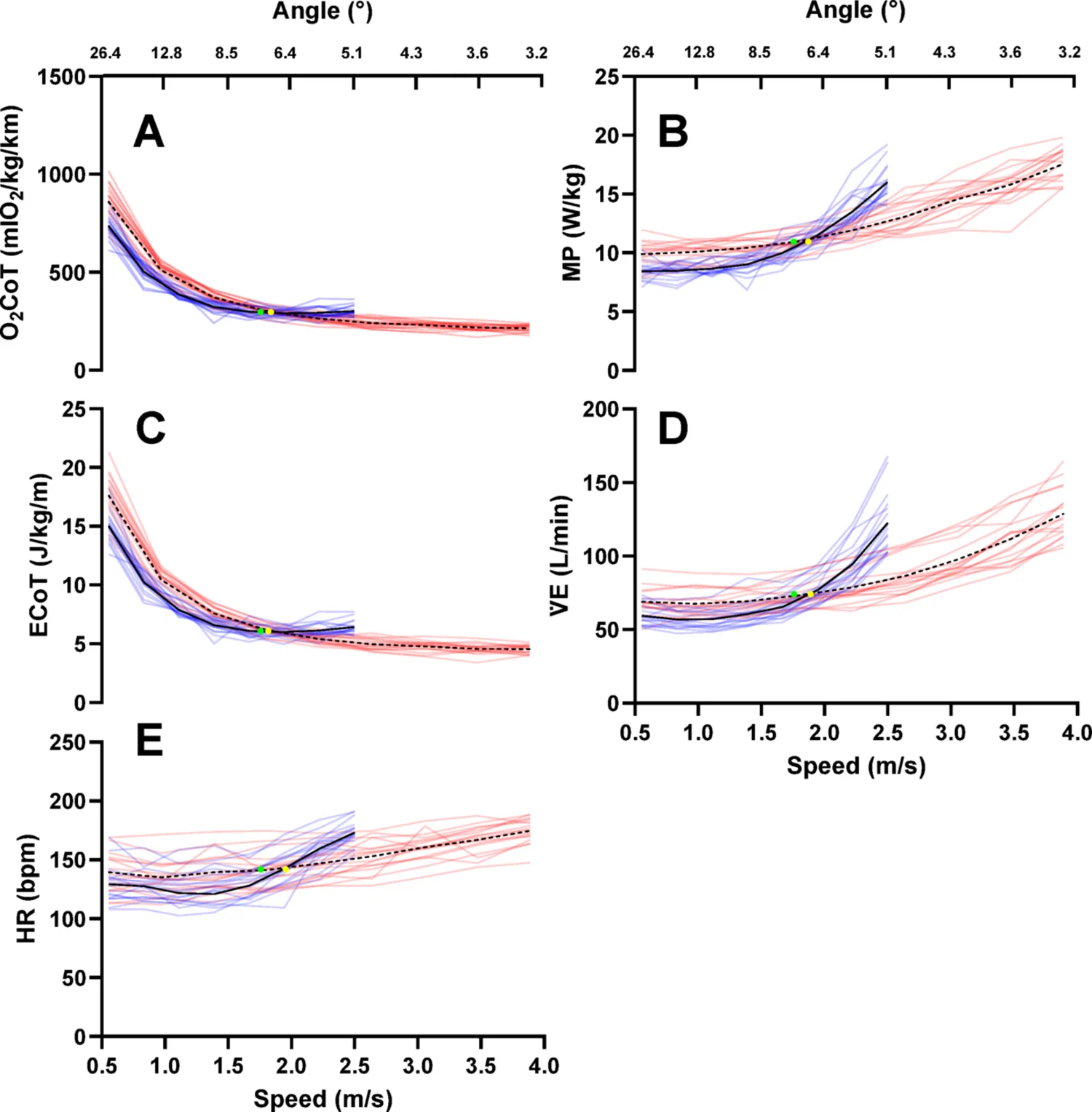
Individual data for each participant are shown for **A** oxygen cost of transport (O_2_CoT), **B** Metabolic Power (MP), **C** Energy Cost of Transport (ECoT), **D** Ventilation (V̇E), **E** Heart Rate (HR) during walking (blue line) and running (red line). The continuous black line illustrates the mean for walking and the dotted line the mean for running. Green dots indicate the mean Preferred Transition Speed (PTS) and yellow dots reflect the Optimal Transition Speed (OTS). All variables are plotted as a function of speed (m·s⁻¹) on a common X-axis and a second non-linear X-axis above for slope angle (°)

**Fig. 2:**
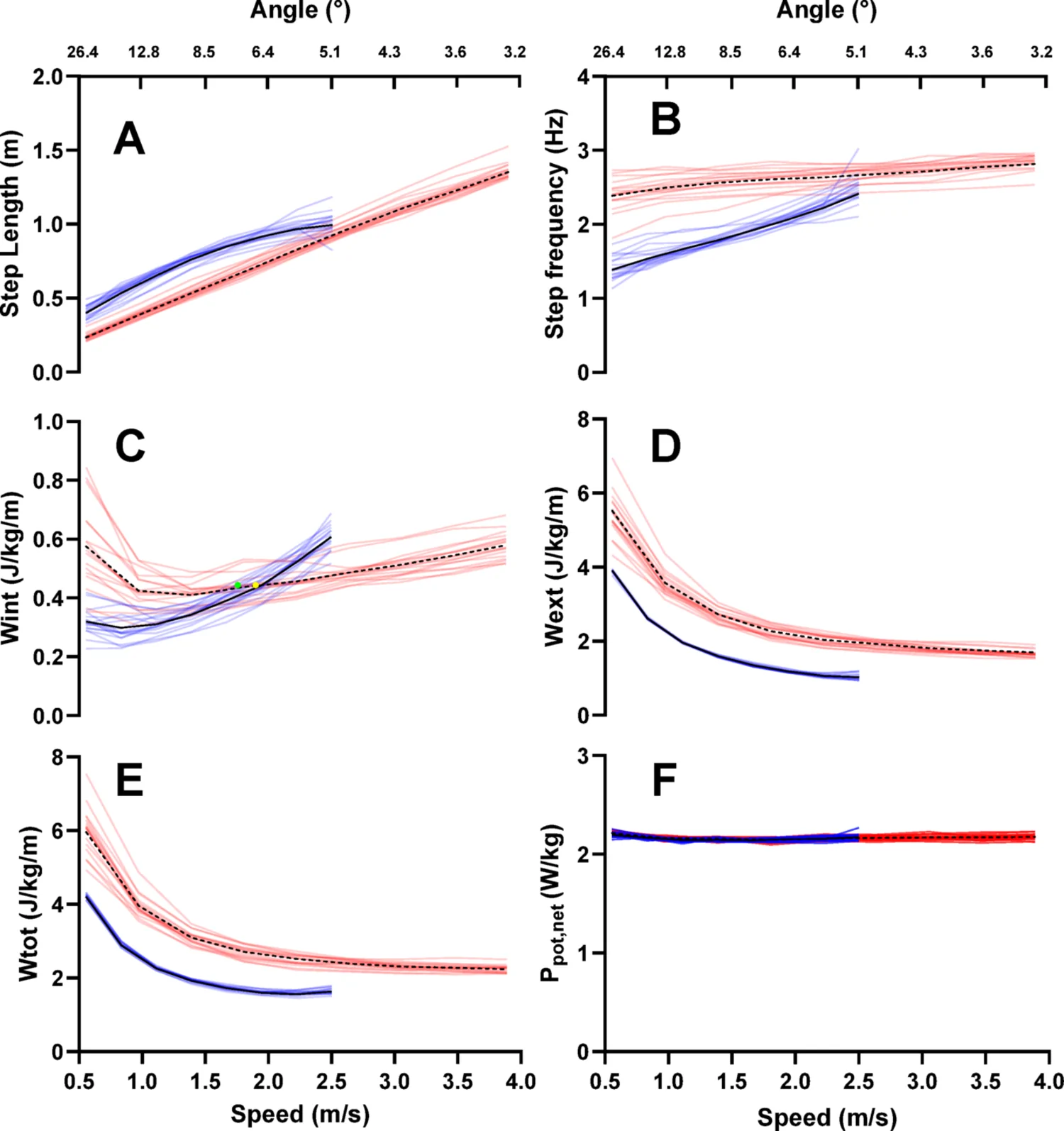
Step length (**A**) is expressed in meters (m), and step frequency (**B**) in Hertz (Hz) Internal Mechanical Work (**C**) (Wint), External Mechanical Work (**D**) (Wext), and Total Mechanical Work (**E**) (Wtot) are presented in J·m⁻¹·kg⁻¹. Net potential power (**F**) (P_pot, net_) is presented in W·kg⁻¹. Green dots indicate the mean Preferred Transition Speed (PTS) and, where applicable, yellow dots reflect the Optimal Transition Speed (OTS). The continuous black line illustrates the mean for walking and the dotted line the mean for running. All variables are plotted as a function of speed (m·s⁻¹) on a common X-axis and a second non-linear X-axis above for slope angle (°)

The mean PTS of the participants was 1.76 [1.66;1.84] m·s⁻¹, which corresponded to an angle of 7.3°. Physiological variables are presented in Fig. 1, while biomechanical variables are presented in Fig. 2.

**Fig. 3:**
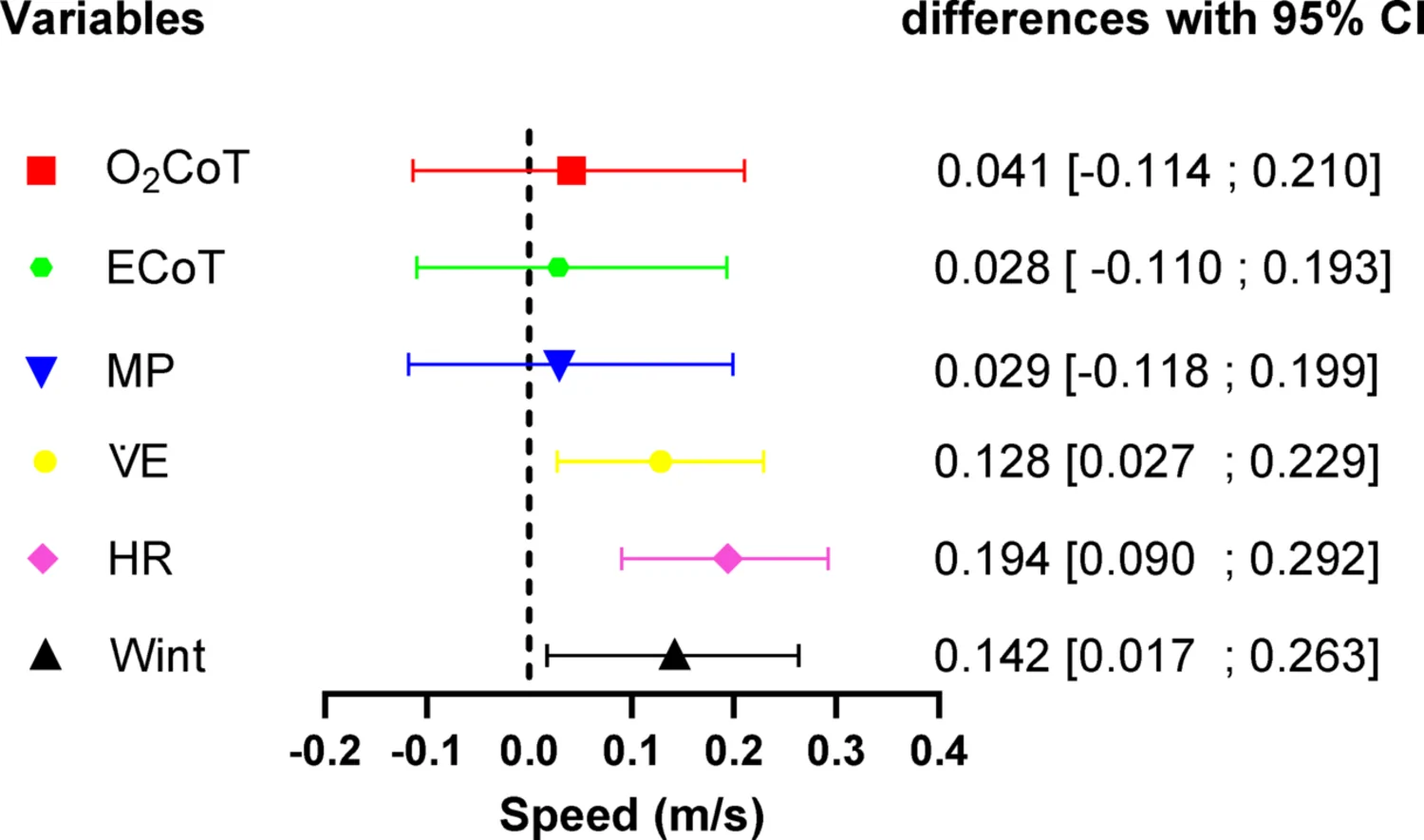
Mean differences between the optimal transition speed for each variable and the preferred transition speed, with 95% confidence intervals. Red square represents the oxygen Cost of Transport (O₂CoT). The green hexagon represents the energetical cost of Transport (ECoT). Blue triangle is the metabolic power (MP), while the yellow square is the ventilation (V̇E). Heart rate (HR) is represented by the rose rhombus and finally Internal mechanical work (Wint) is illustrated by the black triangle. A difference was considered statistically significant if the CI did not include zero

O_2_CotOTS (Fig. 1.A) was at 1.80 [1.69; 1.91] m·s⁻¹ with a mean difference with PTS of 0.041 m·s⁻¹, 95% CI [−0.11; 0.21] showing a non-significant difference with PTS (Fig. 3). MPOTS (Fig. 1.B) was at 1.79 m·s⁻¹, 95% CI [1.74; 1.94], with a mean difference with PTS of 0.029 m·s⁻¹, 95% CI [−0.11; 0.19], revealing no significant difference (Fig. 3). ECoTOTS (Fig. 1.C) was at 1.79 m·s⁻¹, 95% CI [1.69; 1.89], with a mean difference with PTS of 0.02 m·s⁻¹, 95% CI [–0.11; 0.19], suggesting no significant difference between PTS and ECoTOTS (Fig. 3). V̇EOTS (Fig. 1.D) was at 1.89 m·s⁻¹, 95% CI [1.84; 1.94], with a mean difference with PTS (Fig. 3) of 0.13 m·s⁻¹, 95% CI [0.03; 0.23], showing a significant difference from PTS. HROTS (Fig. 1.E) took place at 1.95 m·s⁻¹, 95% CI [1.87; 2.03], and the mean difference with PTS (Fig. 3) was 0.19 m·s⁻¹, 95% CI [0.09; 0.29], demonstrating a statistically significant difference.

Step length (Fig. 2.A) varied from 0.39 m to 0.99 m for walking and from 0.23 m to 1.35 m for running and no OTS was observed for this variable either. Step frequency (Fig. 2.B) varied from 1.38 Hz to 2.41 Hz and 2.38 Hz to 2.81 Hz for walking and running, respectively. No OTS was identified in the range of investigation for this variable. WintOTS (Fig. 2.C) was at 1.90 m·s⁻¹, 95% CI [1.78; 2.02], with a mean difference with PTS of 0.14 m·s⁻¹, 95% CI [0.01; 0.26]. This indicates a statistically significant difference (Fig. 3) with PTS. Wext varied from 2.61 to 1.02 J·kg⁻¹·m⁻¹ and from 5.52 J·kg⁻¹·m⁻¹ to 1.69 J·kg⁻¹·m⁻¹ for walking and running, respectively. No OTS was observed (Fig. 2.D). Wtot varied from 4.20 J·kg⁻¹·m⁻¹ to 1.63 J·kg⁻¹·m⁻¹ and from 5.96 J·kg⁻¹·m⁻¹ to 2.23 J·kg⁻¹·m⁻¹ during walking and running, respectively. No OTS was observed for this variable as well (Fig. 2.E). As expected, P_pot, net_ (Fig. 2.F) varied between 2.19 and 2.15 W·kg⁻¹ for both running and walking and did not differ between conditions or locomotion modalities.

## Discussion

This study examined the preferred transition speed (PTS) during uphill locomotion performed at a constant vertical speed (800 m·h⁻¹). It also assessed which physiological and mechanical variables yield crossover-based OTS that align with PTS. Overall, PTS aligned most closely with energetic crossover criteria, whereas HR, V̇E and Wint crossovers occurred at higher speeds and other mechanical descriptors did not yield a crossover within the tested range.

The PTS occurred at 1.76 m·s⁻¹, corresponding to a 7.2° slope at a vertical speed of 800 m·h⁻¹. This estimate was closely aligned with published predictions despite differences in protocol design. When Brill and Kram’s regression model (Brill and Kram 2021) was expressed at a vertical speed of 800 m·h⁻¹, the predicted PTS was 1.70 m·s⁻¹ (− 3% vs. the measured value), whereas extrapolation from Minetti et al. (Minetti et al. 1994a) yielded 1.84 m·s⁻¹ (+ 5%). These small deviations, within the range expected from inter-study differences in instrumentation, modeling, and inter-individual variability, support the consistency of the PTS obtained under iso-$\:{V}_{vert}$ constraints for 800 m·h⁻¹.

**Fig. 4:**
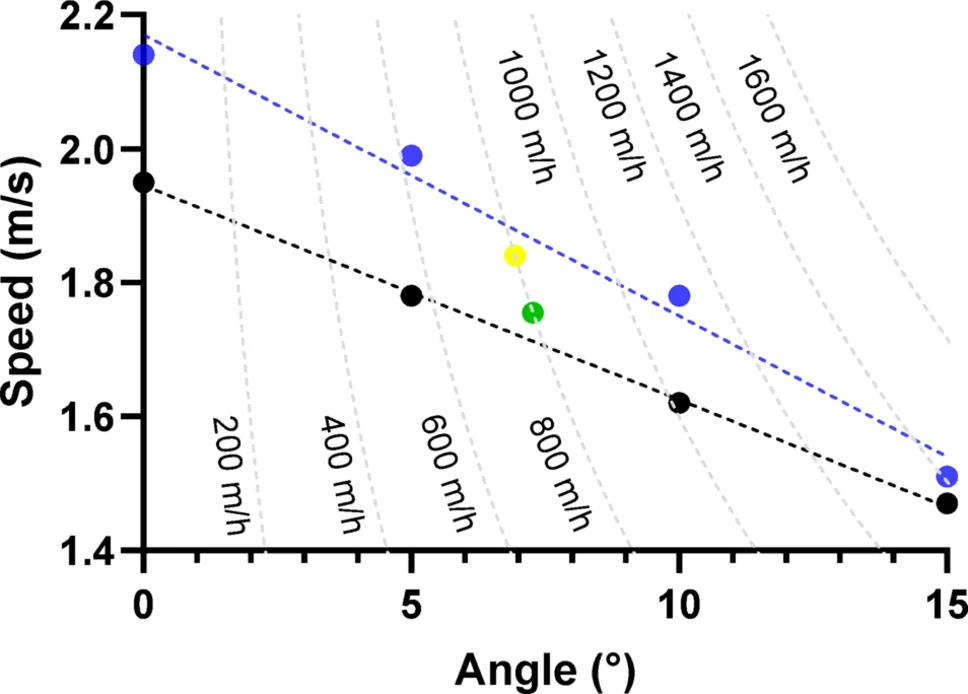
Reconstruction of Brill and Kram’s grade-dependent relationships between metabolic power optimal transition (MPOTS) and spontaneous transition speed (PTS). Blue circles (and blue dotted line) show Brill and Kram’s MPOTS across grades. Black circles (and black dotted line) show their measured PTS obtained using fixed-grade, incremental/decremental protocols. Yellow circle indicates the PTS measured in the present iso-$\:{V}_{vert}$ protocol ($\:{V}_{vert}$ = 800 m·h⁻¹; incline = 7.2°), and the green circle indicates the corresponding MPOTS from the present study. Grey dashed curves represent iso-vertical-speed contours (200–1600 m·h⁻¹) in slope–speed space

Beyond the absolute PTS value, the present results indicate a distinct relationship between PTS and energetic crossover criteria. ECoTOTS, O₂CoTOTS and MPOTS did not differ from PTS, indicating that the preferred transition occurred near the energetic walk–run crossover. This contrasts with Minetti et al. (5), who reported PTS values slower than O₂CoTOTS up to 8.5°, and with Brill and Kram (Brill and Kram 2021), who reported an MPOTS–PTS separation up to 15°. Because ECoT is metabolic power normalized by speed, convergence between ECoTOTS and MPOTS is expected when OTS is defined as a walk–run crossover in speed; the key observation here is the additional alignment of these crossover-based OTS with PTS under an iso-$\:{V}_{vert}$ constraint. Overlaying PTS and MPOTS on a reconstruction of Brill and Kram’s grade-dependent relationships (Fig. 4) illustrates that their reported PTS and OTS do not occur at the same vertical speed, whereas the present protocol constrained transitions to a single iso-$\:{V}_{vert}$ contour (800 m·h⁻¹). Under fixed-grade incremental/decremental designs (Brill and Kram 2021), vertical speed varies substantially as belt speed changes, whereas holding vertical speed constant standardizes the net gravitational potential power requirement across the tested slope–speed combinations. This constraint does not fix metabolic demand, but it couples speed and slope (lower belt speed at steeper grades), which can reorganize spatiotemporal patterning (e.g., step length and step frequency) and mechanical work distribution along the iso-$\:{V}_{vert}\:$contour. This coupling may therefore influence the degree of PTS–OTS dissociation reported under fixed-grade protocols, even when the resulting PTS remains close to literature-based predictions. A within-subject comparison of iso-grade and iso-$\:{V}_{vert}$ protocols is required to test this hypothesis, given the known inter- and intra-individual variability of walk–run and run–walk transitions (32). Testing higher vertical speeds may further clarify protocol dependence, as slope–speed coupling becomes more extreme.

Wint was the only biomechanical parameter to show a crossover between walking and running, but WintOTS was significantly different from PTS. This mismatch argues against a simple “internal-work trigger” explanation of the transition under the present conditions. Importantly, Wint is strongly speed-dependent and does not behave similarly in walking and running, as walking Wint increases more steeply with speed than running Wint (Minetti et al. 1994b; Minetti 1998). This gait-specific speed dependency may add uncertainty when interpreting Wint across gradients, particularly when slope-induced changes in speed, stride frequency, and limb segment motion occur simultaneously. Previous gradient locomotion studies also suggest that Wint values and their relative contribution to Wtot depend on gait, speed, gradient, and methodological choices (Minetti and Ardigò 2001; Rasica et al. 2020). Therefore, although the Wint crossover observed in the present study is biomechanically relevant, it should be interpreted cautiously and does not by itself support Wint as a primary determinant of PTS.

The present findings also showed a significant difference between PTS and HROTS. Brill and Kram (Brill and Kram 2021) reported poor correlations between HR-based and preferred transitions across inclines, with protocol-dependent differences across grades. The present result supports their conclusion. V̇EOTS and PTS were significantly different, indicating that a ventilation-based crossover does not provide a reliable marker of the preferred walk–run transition under the tested conditions. Limited data are available on ventilatory crossover behavior on inclines, but this outcome is consistent with level-ground observations showing that ventilatory variables do not necessarily yield stable gait-transition thresholds (Mercier et al. 1994). Importantly, the absence of alignment between PTS and step-frequency behavior further supports this interpretation: locomotor–respiratory coupling is often observed during walking and running, with breathing rhythm tending to entrain to stride rhythm at preferred ratios, and experimental manipulations of stride frequency have been shown to influence ventilation (Bertram 2015; Selinger et al. 2015, p. 201). Further breathing-pattern analyses also indicated substantial inter-individual variability (e.g., inconsistent crossovers in respiratory frequency), which likely reflect different ventilatory strategies (frequency vs. tidal-volume adjustments) in response to combined metabolic and biomechanical constraints.

Taken together, these findings can be framed within an “energetic landscape” perspective: on level ground, preferred locomotor behaviors are often interpreted as emerging from minimization of energetic cost per distance, and humans can adapt gait parameters to move toward energetically favorable solutions (Bertram 2015; Selinger et al. 2015). Although these demonstrations were obtained on the level, the present uphill data suggest that a related optimization principle may also influence gait choice when the task is defined by a fixed ascent rate. In particular, the alignment between PTS and energetic crossover criteria indicates that the walk–run transition under iso-$\:{V}_{vert}$ condition occurs near the point where walking and running impose comparable energetic demands for the same climbing requirement. This interpretation emphasizes that, in steep uphill locomotion, the relevant “currency” for decision-making may be the energetic demand to gain elevation (or equivalently the metabolic power required at a given vertical velocity), rather than a ventilatory or heart-rate crossover. At the same time, energetic minimization is unlikely to be the sole constraint, as locomotor choices may also reflect trade-offs that reduce local muscle overloading and fatigue (MacDonald et al. 2022), which could contribute to the weak alignment of cardiorespiratory crossovers with PTS. From an applied standpoint, these results support an economy-centered approach to uphill gait management: quantifying walking and running economy around competition-relevant ascent rates may help identify the individual slope–speed region where energetic crossover occurs and where transitions are most likely. Interventions that improve economy in both gaits near this region (technique, strength-endurance, or task-specific uphill training) may therefore represent a practical lever to influence transition behavior and climbing efficiency, consistent with energetics-based interpretations of preferred speeds in natural settings (Selinger et al. 2022).

## Conclusions

At a constant vertical speed of 800 m·h⁻¹, the PTS appeared to be governed primarily by a metabolic-saving strategy, with no evidence that internal mechanical work or any mechanical variables measured influenced the transition. These findings support the view that, even during uphill locomotion, PTS reflects a strategy aimed at minimizing the energetic cost of transport and metabolic power rather than being dictated by mechanical constraints.

## Data Availability

The datasets generated and/or analysed during the current study are available from the corresponding author on reasonable request.

## Abbreviations

AICc: Small-sample corrected Akaike Information Criterion
CoM: Centre of mass
ECoT: Energy cost of transport
HR: Heart rate
MP: Metabolic power
O₂CoT: Oxygen cost of transport
OTS: Optimal transition speed
Ppot,net: Net potential power
PTS: Preferred transition speed
V̇E: Minute ventilation
Wext: External mechanical work
Wint: Internal mechanical work
Wtot: Total mechanical work
